# Association between choroidal microvasculature in the eye and Alzheimer's disease risk in cognitively healthy mid‐life adults: A pilot study

**DOI:** 10.1002/dad2.70075

**Published:** 2025-01-16

**Authors:** Jamie Burke, Samuel Gibbon, Audrey Low, Charlene Hamid, Megan Reid‐Schachter, Graciela Muniz‐Terrera, Craig W. Ritchie, Baljean Dhillon, John T. O'Brien, Stuart King, Ian J. C. MacCormick, Thomas J. MacGillivray

**Affiliations:** ^1^ Robert O Curle Ophthalmology Suite Institute for Regeneration and Repair University of Edinburgh Edinburgh UK; ^2^ School of Mathematics, University of Edinburgh Edinburgh UK; ^3^ Centre for Clinical Brain Sciences Chancellor's Building Edinburgh UK; ^4^ Department of Psychiatry University of Cambridge Cambridge UK; ^5^ Edinburgh Imaging, The Queen's Medical Research Institute, University of Edinburgh Edinburgh UK; ^6^ Heritage College of Osteopathic Medicine Ohio University Athens Ohio USA; ^7^ Princess Alexandra Eye Pavilion Chalmers Centre Edinburgh UK; ^8^ Institute for Adaptive and Neural Computation University of Edinburgh Edinburgh UK

**Keywords:** apolipoprotein E ε4, choroid, dementia, optical coherence tomography, retina

## Abstract

**INTRODUCTION:**

We explored associations between measurements of the ocular choroid microvasculature and Alzheimer's disease (AD) risk.

**METHODS:**

We measured the choroidal vasculature appearing in optical coherence tomography (OCT) scans of 69 healthy, mid‐life individuals in the PREVENT Dementia cohort. The cohort was prospectively split into low‐, medium‐, and high‐risk groups based on the presence of known risk factors (apolipoprotein E [*APOE*] ε4 genotype and family history of dementia [FH]). We used ordinal logistic regression to test for cross‐sectional associations between choroidal measurements and AD risk.

**RESULTS:**

Choroidal vasculature was progressively larger between ordinal risk groups, and significantly associated with risk group prediction. *APOE* ε4 carriers had thicker choroids and larger vascularity compared to non‐carriers. Similar trends were observed for those with a FH.

**DISCUSSIONS:**

Our results suggest a potential link between the choroidal vasculature and AD risk. However, these exploratory findings should be replicated in a larger sample.

**Highlights:**

- Ocular choroidal microvasculature is of interest in relation to neurodegeneration due to its autonomic response to systemic, pathophysiological change.
- Choroidal changes in the prodromal stage of Alzheimer's disease (AD) are unexplored.
- The PREVENT Dementia cohort offers a unique, non‐invasive study of the microvasculature in mid‐life individuals at increased risk for developing AD.
- Significantly increased ocular choroidal vasculature was associated with increased risk (apolipoprotein E carrier and/or family history of dementia) for AD.
- These exploratory results suggest a potential association between the ocular choroidal vasculature and AD risk. However, findings should be replicated in a larger sample.

## 1 INTRODUCTION

Non‐invasive examination of the back of the eye using optical coherence tomography (OCT) is increasingly used to investigate neurodegenerative diseases, including Alzheimer's disease (AD),^1^ which has a strong vascular component.^2^ While the primary goal of OCT has often been to image the cross‐sectional retinal layers (a neuronal measure and marker of neurodegeneration), recent advances in enhanced depth imaging OCT (EDI‐OCT) ^3^ permit visualization of the choroid, a dense vascular mesh posterior to the retina, which provides essential maintenance to the photoreceptors.^4^ Compared to the retinal circulation, the choroidal circulation has significantly greater blood flow and perfusion pressure,^5^, ^6^ and is innervated by the central autonomic network.^7^ Autonomic dysfunction is common in adults with mild cognitive impairment,^8^ and in those already living with dementia,^9^ which makes the choroid a prime candidate for investigating pathophysiological response in relation to these conditions. Since the advent of EDI‐OCT, there have been numerous studies linking choroidal changes to both systemic health outcomes^10^ and neurological diseases.^11^, ^12^, ^13^

However, there have been conflicting reports of choroidal changes in established AD in older populations,^14^, ^15^, ^16^, ^17^, ^18^, ^19^, ^20^ raising questions about the underlying mechanisms and stages of the disease that might influence the choroidal vasculature. One possibility is that genetic factors, such as the apolipoprotein E (*APOE*) ε4 allele, and family history of dementia (FH), both of which have been identified as important determinants of AD risk,^21^, ^22^, ^23^ may play a role. To our knowledge, only one study directly assessed the relationship between choroidal measures and genetic risk (*APOE* ε4) in cognitively normal, old‐age individuals, but found no evidence of significant differences between carriers and non‐carriers.^24^ Choroidal changes in the preclinical or prodromal stage of AD remain unexplored and could provide valuable insights into the underlying mechanisms linking vascular pathology to prospective cognitive decline.

The PREVENT Dementia study,^25^ investigates and tracks concurrent cerebral and retinal vascular changes in a cohort of mid‐life individuals, approximately half of whom are at increased risk of AD, owing to FH. While most research on ocular microvascular changes in AD focuses on older age groups,^14^, ^15^, ^16^, ^17^, ^18^, ^19^, ^24^ the PREVENT Dementia cohort presents a unique opportunity to examine the microvasculature in individuals who may be in the very early preclinical stage of disease.^26^ Accordingly, we conducted an exploratory, pilot study to explore the associations between four choroidal measures (choroidal thickness, total choroidal area, choroidal vascularity index [CVI], total vessel area) and two genetic risk factors (*APOE* ε4, FH) in cognitively healthy mid‐life adults at baseline of this study cohort.

## 2 METHODS

### 2.1 Study participants

The protocol for the PREVENT Dementia study study has been outlined previously.^25^, ^27^ It aimed to recruit participants aged between 40 and 59 from five sites in the UK and Ireland, and to include a significant portion with a FH (≈ 50%). Ophthalmic imaging was a sub‐study, conducted exclusively at the Edinburgh site, which participants could opt into after cerebrovascular assessment. Participants provided written informed consent and the study adhered to the principles of the Declaration of Helsinki.

Eligibility criteria for the Edinburgh arm was competent and consenting adults recruited from the wider PREVENT Dementia study study who had the capacity to maneuver themselves to the retinal imaging machines unaided and follow instructions to facilitate patient fixation during imaging at baseline. Exclusion criteria were those with a clinical diagnosis of dementia, those without the capacity to consent at baseline, or individuals not able to fully understand written and verbal English. Exclusion criteria also included participants with current or previous ocular disease affecting the retina or choroid such as glaucoma, macular degeneration, diabetic retinopathy, uveitis, vitreous hemorrhage, pachychoroid, pathological myopia, ischemic optic neuropathy, optic neuritis or other optic nerve diseases, or those who had undergone previous ocular surgery such as cataract surgery or retinal surgery. These were self‐reported at the point of acquisition and any incidental findings were referred to an ophthalmologist for confirmation and subsequent referral.

### 2.2 Image capture

OCT was captured in both eyes using the Heidelberg spectral domain SPECTRALIS Retina HRA+OCT Module (Heidelberg Engineering). Fovea‐centered OCT scans of the retina and choroid (Figure S1 in supporting information) were taken using active eye tracking to prevent artifacts from eye movement, covering a 30° angle at high speed, resulting in ≈ 9 mm field of view in the transverse direction. A single, horizontal‐line B‐scan was taken with an automatic real time (ART) value of 100 (the number of averaged B‐scans taken during a single acquisition) to reduce speckle noise. Figure S1 shows a typical OCT capture with the en face localizer scanning laser ophthalmoscopy (SLO) image in panel (A) and the B‐scan in panel (B). B‐scans with a signal‐to‐noise quality index (provided in the HeyEx viewer software, version 1.10.4.0; Heidelberg Engineering) of < 15 dB were excluded, according to the OSCAR‐IB criteria^28^ for retinal OCT quality assessment.

### 2.3 Choroidal measurements

**FIGURE 1:**
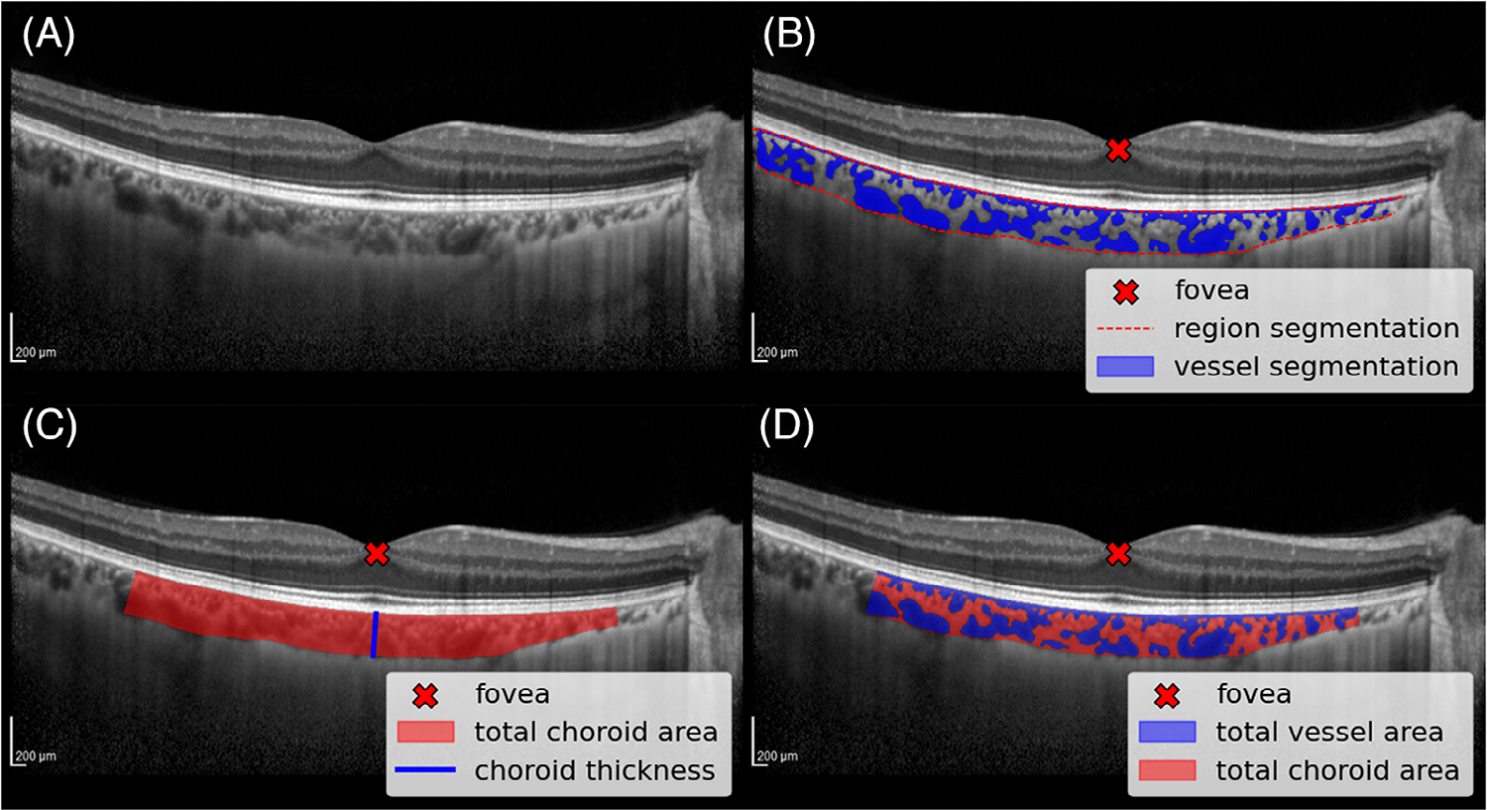
Output from Choroidalyzer and computation of choroidal measurements in a fovea‐centered region of interest. A, OCT B‐scan. B, OCT B‐scan with output segmentations from applying Choroidalyzer. C, Derivation of total choroid area and subfoveal choroid thickness in a prescribed ROI. D, Derivation of total vessel area and CVI in a prescribed ROI. CVI, choroidal vascularity index; OCT, optical coherence tomography.

Measurements of fovea‐centered subfoveal choroidal thickness, total choroidal area, total vessel area, and CVI were computed using Choroidalyzer,^29^ a fully automatic deep learning‐based image analysis pipeline. Briefly, Choroidalyzer automatically segments the choroid region and vasculature of an OCT B‐scan (Figure 1A) and detects the fovea (Figure 1B). The choroid was defined as the vascular space between the hyperreflective Bruch's membrane (anterior) and sclera (posterior). Choroidal thickness was measured as a straight‐line micron distance underneath the fovea, locally perpendicular to Bruch's complex (Figure 1C, blue). The total choroidal area was calculated as the number of pixels within a fovea‐centered region of interest (ROI), converted into mm^2^ (Figure 1C, red). Similarly, total vessel area was calculated as the number of vessel pixels within the prescribed ROI (Figure 1D, blue), also converted into mm^2^. CVI measures the proportion of vasculature within the choroid and is a dimensionless ratio of vessel pixels to choroid pixels (Figure 1D, blue:red), similarly, measured in a fovea‐centered ROI. Subsequent region, vessel, and fovea detection was checked manually (author J.B.) before measurements were computed. Choroid measurements were made for three distinct ROI's defined by: 0.5, 1.5, and 3 mm distance from either side of the fovea, following the Early Treatment Diabetic Retinopathy Study (ETDRS) grid.^30^ The software is implemented in Python (version 3.11.6).

**RESEARCH IN CONTEXT**

1. **Systematic review**: We reviewed the literature on the relationship between ocular choroidal microvasculature and Alzheimer's disease (AD). While the ocular choroid has been studied in onset of AD at old age using non‐invasive optical coherence tomography (OCT), changes in choroidal vasculature in the preclinical or prodromal stage of AD at mid‐life remain relatively unexplored.
2. **Interpretation**: In a mid‐life cohort of asymptomatic individuals from the PREVENT Dementia cohort, we found significantly enlarged choroidal vasculature in the high‐risk groups (both an apolipoprotein E ε4 carrier and family history of dementia). This could be indicative of autonomic dysfunction, suggesting a potential link between the choroidal vasculature and AD risk, further supporting evidence of non‐invasive OCT informing on brain health.
3. **Future directions**: Future research should confirm these associations in larger, more diverse populations. Additionally, longitudinal studies are needed to see if the choroidal microvasculature may be predictive of developing future cognitive decline and later life AD.

### 2.4 Data exploration and statistical analysis

Participants in the PREVENT Dementia cohort^25^ were stratified into three risk groups based on *APOE* ε4 presence and FH by design: “high” risk was defined when both *APOE* ε4 and FH were present, “medium” risk when either *APOE* ε4 or FH were present, and “low” risk when neither were present.

As this was an exploratory study with a relatively small sample size, our primary objective was to report descriptive statistics and investigate potential trends and associations. We first graphed choroidal measurements, testing for normality using the Shapiro–Wilk test, and performed univariate hypothesis testing on the mean differences between choroidal measures stratified for each risk factor. Alongside univariate statistical tests with individual markers, we assumed a biological ordering between risk groups and used ordinal logistic regression with each choroidal measure in turn as a single covariate in an unadjusted model, as well as in adjusted models controlling for age, sex, and mean arterial blood pressure. We selected these three covariates based on existing literature.^31^, ^32^, ^33^, ^34^ Additionally, we included scanning metadata parameters OCT scan quality index (a measure of signal‐to‐noise ratio) and OCT scan focus (an approximate measure of refractive error) as covariates, to account for differences in image quality and eye shape. Unadjusted models were estimated so as to retain maximum statistical power in a relatively low sample size.

To minimize the risk of spurious associations from analyses of multiple regions of interest per eye, we only selected choroidal measurements from horizontal‐line scans with a 1.5 mm fovea‐centered ROI, with a preference for the left eye. The left eye tends to have a thinner choroid than the right,^35^ helping overcome issues with image quality from optical signal degradation. When the left eye was not available, the right eye was used if available. Any missing values were removed at the point of analysis (see Table 1). Analyses were performed using Python's statsmodels (version 0.14.0).

**TABLE 1:** Demographics and study variables, stratified by risk group.

	Risk group				
	Low	Medium	High	Overall	*p* value
**Participants**	26	26	17	69	
**Age** (years)	50.6 (6.3)	52.4 (4.5)	51.6 (6.4)	51.6 (5.7)	0.526^*^
**Sex** (female)	14 (53.9%)	17 (65.4%)	10 (58.8%)	41 (59.4%)	0.697^**^
**Scan focus** (diopters)	0.75 (2.40)	−0.53 (2.52)	−0.68 (1.50)	−0.75 (2.40)	0.770^*^
**Scan quality** (dB)	32.5 (3.3)	33.0 (3.7)	34.3 (3.1)	33.2 (3.4)	0.238^*^
**Hypertension**	4 (15.4%)	3 (11.5%)	1 (6.3%)	8 (11.8%)	0.671^**^
**Blood pressure** (bpm)	138.6 (13.0)	124.7 (16.4)	127.4 (13.7)	130.6 (15.7)	**0.003** ^*^
**BMI** (kg/m^2^)	30.4 (6.9)	28.1 (5.3)	28.7 (4.2)	29.1 (5.7)	0.366^*^
**Smoking**					0.445^**^
Current	1 (3.8%)	1 (3.8%)	0 (0%)	2 (2.9%)	
Ex	8 (30.8%)	7 (26.9%)	9 (52.9%)	24 (34.8%)	
Non	17 (65.4%)	18 (69.2%)	8 (47.1%)	43 (62.3%)	
**Diabetes**	1 (3.8%)	2 (7.7%)	0 (0%)	3 (4.3%)	0.475^**^
**Risk factors**					
*APOE* ε4 status	0 (0%)	8 (30.8%)	17 (100.0%)	25 (36.2%)	**<0.001** ^**^
Family history	0 (0%)	18 (69.2%)	17 (100.0%)	35 (50.7%)	**<0.001** ^**^
**Choroidal measures**					
Total choroid area (mm^2^)	0.68 (0.25)	0.71 (0.19)	0.87 (0.30)	0.74 (0.26)	**0.044** ^*^
Choroid thickness (µm)	250.39 (84.48)	257.00 (78.37)	303.35 (105.56)	265.93 (89.32)	0.133^*^
CVI	0.48 (0.06)	0.53 (0.07)	0.53 (0.08)	0.51 (0.07)	**0.007** ^*^
Total vessel area (mm^2^)	0.31 (0.14)	0.36 (0.14)	0.46 (0.18)	0.37 (0.16)	**0.008** ^*^

*Note*: All values are *N* (%) or Mean (standard deviation). Although the modeling below only controlled for age, sex, blood pressure, OCT scan focus and image quality, we report other cardiovascular risk factors for completeness. Group‐based *p* values < 0.05 are in bold type. Missing data (number of eyes, percentage): Hypertension (1, 1.5%); BMI (2, 2.9%); CVI (2, 2.9%); Vessel area (2, 2.9%).

Abbreviations: *APOE*, apolipoprotein E; BMI, body mass index; CVI, choroidal vascularity index.

* One‐way analysis of variance.

** Chi squared.

## 3 RESULTS

**FIGURE 2:**
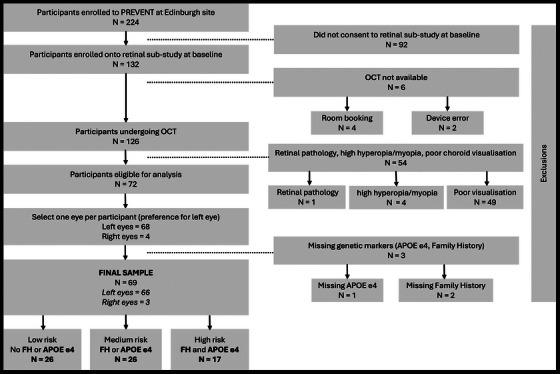
Sample derivation flowchart. *APOE*, apolipoprotein E; FH, family history; OCT, optical coherence tomography; PREVENT, Presymptomatic Evaluation of Experimental or Novel Treatments for Alzheimer's Disease.

The Edinburgh site recruited 224 participants, of which 132 provided written informed consent to the retinal sub‐study at baseline. Of these 132 participants, 126 underwent successful OCT capture (device error, *N* = 2; room booking issues, *N* = 4). Participants with retinal pathology observed at the point of acquisition (*N* = 1, macular degeneration) were excluded from our analysis to prevent abnormal retinae from confounding our results, as well as highly myopic/hyperopic eyes (*N* = 4, refractive error > ± 6 diopters, approximately measured using the OCT scan focus on the imaging device),^36^ which have a known association with the choroid.^37^ Only EDI‐OCT scans with sufficient choroid visualization were selected for analysis (*N* = 72). After removing individuals with missing data for *APOE* ε4 and FH (*N* = 3), the final sample contained 69 participants (66 left eyes, 3 right eyes), stratified into low‐ (*N* = 26), medium‐ (*N* = 26), and high‐ (*N* = 17) risk groups. A sample derivation flowchart is presented in Figure 2. While we cannot be certain of no selection bias in our final sample, comparing any distribution differences between the final sample (*N* = 69) and the remaining cohort (*N* = 155), there was no evidence of significant differences in demographic or cardiovascular variables (Table S1 in supporting information).

Demographics and study variables are summarized in Table 1. The mean age was 51.6 years (standard deviation [SD] = 5.71) with a slight female majority (*N* = 41, 59.4%). Mean arterial blood pressure was slightly higher in the low‐risk group compared to the medium‐ and high‐risk groups (*p* = 0.001, *p* = 0.01, respectively). No other evidence of significant differences in demographic or cardiovascular variables were observed between risk groups.

**FIGURE 3:**
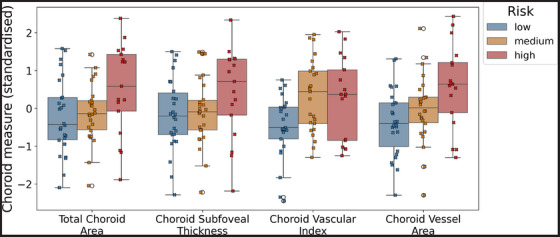
Boxplots showing the relationship between choroidal measures and risk groups, with individual datapoints overlaid in a swarm‐plot.

Choroidal measurements were normally distributed (Figure S2 in supporting information). Figure 3 presents grouped boxplots of choroidal measurements and prospectively defined AD risk groups, showing a biological gradient between increasing risk and increasing choroidal measurements, particularly for choroid vessel area.

After adjusting for age, sex, blood pressure, OCT scan focus, and OCT image quality, larger choroidal measurements were significantly associated with increased risk for AD. Specifically, after adjusting for demographic, cardiovascular, and scanning parameters, the presence of *APOE* ε4 and/or FH was significantly associated with larger total choroidal area (odds ratio [OR] per SD increase = 1.84 [confidence interval (CI): 1.08–3.16], *p* = 0.026), and total vessel area (OR per SD increase = 2.28 [CI: 1.30–3.99], *p* = 0.004), but significance was not reached with choroidal thickness (OR per SD increase = 1.63 [CI: 0.97–2.73], *p* = 0.064) or CVI (OR per SD increase = 1.63 [CI: 0.97–2.73], *p* = 0.065). These findings were also reflected in the unadjusted models.

The magnitude of effect for total vessel area was the largest of all measures. Put another way, for every unit SD increase in total vessel area (0.16 mm^2^), on average it is 2.28 times more likely that this individual has at least one risk marker for AD (*APOE* ε4, FH) rather than none, after holding all other covariates constant. Results for both adjusted and unadjusted models are summarized in Table 2. Figure S3 in supporting information shows two representative examples from the low‐risk group (Figure S3A) and high‐risk groups (Figure S3B), with the choroid in panel (B) presenting with a distinctively larger choroidal space and vascular tissue than the choroid in panel (A) underneath the fovea (red arrows).

**TABLE 2:** OR (with 95% CI and corresponding *p* values) for each ordinal logistic regression model, predicting risk group with each choroidal measure in turn.

**Choroid measure**	Adjusted models		Unadjusted models	
	OR (CI)	*p*	OR (CI)	*p*
Total choroid area (mm^2^)	1.84 (1.08–3.16)	**0.026**	1.77 (1.09–2.88)	**0.021**
Choroid thickness (µm)	1.63 (0.97–2.73)	0.064	1.54 (0.97–2.47)	0.070
CVI	1.63 (0.97–2.73)	0.065	1.87 (1.16–3.00)	**0.010**
Total vessel area (mm^2^)	2.28 (1.30–3.99)	**0.004**	2.14 (1.30–3.51)	**0.003**

*Note*: Adjusted models include age (standardized), blood pressure (standardized), OCT scan focus (standardized), OCT image quality index (standardized), and sex as covariates. To provide stable confidence intervals in logistic models, all choroidal measures were standardized. *p* values < 0.05 are in bold type.

Abbreviations: CI, confidence interval; CVI, choroidal vascularity index; OCT, optical coherence tomography; OR, odds ratio.

Considering individual risk, total choroidal area and vascular tissue were larger for *APOE* ε4 carriers and FH individuals, and these differences were supported by independent *t* tests for total vessel area (*p* = 0.022, *p* = 0.009, respectively), and for total choroidal area (*p* = 0.020) and CVI (*p* = 0.022) for *APOE* ε4 carriers and FH, respectively (Table 3). Figure S4 in supporting information illustrates these differences using boxplots and showing distributions.

**TABLE 3:** Distribution of choroidal measures by dementia risk with *p* values for *t* tests.

	*APOE* ε4 carrier status		
	Non‐carrier	Carrier	*p* value
***N***	47	25	
Total choroid area (mm^2^)	0.67 (0.23)	0.83 (0.27)	**0.02**
Choroid thickness (µm)	248.40 (85.36)	288.64 (94.13)	0.081
CVI	0.50 (0.06)	0.53 (0.08)	0.104
Total vessel area (mm^2^)	0.33 (0.14)	0.43 (0.18)	**0.022**

	FH		
	None	Yes	*p* value
***N***	34	36	
Total choroid area (mm^2^)	0.69 (0.23)	0.79 (0.28)	0.122
Choroid thickness (µm)	252.03 (78.16)	283.50 (99.83)	0.146
CVI	0.49 (0.07)	0.53 (0.07)	**0.022**
Total vessel area (mm^2^)	0.32 (0.15)	0.42 (0.16)	**0.009**

*Notes*: *p* values lower than 0.05 are in bold type.

Abbreviations: *APOE*, apolipoprotein E; CVI, choroidal vascularity index; FH, family history of dementia.

## 4 DISCUSSION

We explored associations between the choroidal vasculature and two genetic risk factors for AD (*APOE* ε4, FH) in a mid‐life cohort. We observed a significant, positive association between total choroid area and total vessel area with degree of combined risk. To our best knowledge, this is the first exploration of the choroidal vasculature in cognitively healthy mid‐life adults at increased risk of AD. Previous studies have focused only on retinal differences between genetic risk groups for AD.^38^

The *APOE* ε4 variant is a major risk factor for AD,^23^ and has been associated with a wide range of negative health‐related outcomes or features including cardiovascular disease,^39^ blood–brain barrier dysfunction,^40^ inflammation,^41^ and autonomic dysfunction.^42^ Lohman et al.^42^ reports that *APOE* ε4 carriers exhibit central autonomic dysfunction in early‐stage AD, including the parasympathetic control of cardiovascular functions.^43^ Collins et al.^8^ found that mild cognitively impaired patients were on average 5.6 times more likely to have autonomic dysfunction than controls, showing significant parasympathetic deficits, which may be involved in the pathogenesis of hypotension in dementia. Similarly, given that choroidal blood flow is regulated to some extent by autonomic input, particularly parasympathetic‐mediated vasodilation, we cautiously hypothesize from our exploratory results that parasympathetic deficits may induce vasodilatory, proinflammatory mechanisms or choroidal hypoperfusion. However, the choroid is a highly heterogeneous vascular compartment, and whether larger vasculature corresponds to larger caliber or simply more vessels cannot be fully ascertained with current imaging device resolutions.

We found just one other study that used enhanced‐depth OCT choroid measures to investigate asymptomatic individuals with known *APOE* ε4 status and FH.^24^ However, in contrast to our findings, Ma et al.,^24^ found no evidence of significant differences in choroidal measures between carriers and non‐carriers. One reason for the conflicting findings could be that the Ma et al. study reported choroidal measurements in pixel units. By contrast, we converted choroidal measurements from pixel units into physical units (microns and mm^2^) according to the unique transverse pixel length‐scale each B‐scan corresponds to, so that measurements across the population could be compared more appropriately. Moreover, the average participant age in the Ma et al. study was ≈ 20 years older than ours, suggesting that age differences may also play a role in the contrasting findings.

Enhanced‐depth OCT has previously been used to investigate the link between diagnosed dementia and the choroid, with mixed results. Some have found *smaller* choroidal measurements at the group level in AD and mild cognitively impaired individuals compared to controls.^15^, ^16^, ^17^, ^20^ Bulut et al.^17^ and Gharbiya et al.^16^ showed significant choroidal thinning in AD, but selected both eyes per participant for their statistical analyses without accounting for inter‐eye correlation at the participant level, violating a core statistical principle. Moreover, Gharbiya et al.^16^ and Bayhan et al.^15^ analyzed smaller cohorts and performed measurement of the choroid manually, which has the potential for measurement error, and may be difficult to reproduce.^44^ Recently, Kwapong et al.^20^ found lower choriocapillaris density in OCT‐angiography imaging of early‐age onset AD patients compared to healthy controls and was able to discriminate between such groups. However, compared to the PREVENT Dementia cohort, the Kwapong et al.^20^ cohort was older and already presenting with symptoms sufficient for clinical diagnosis; whether the same approach can be leveraged at the preclinical stage of AD requires further validation.

Other works have observed *larger* choroidal measurements at the group level in AD compared to controls.^18^, ^19^ Asanad et al.^18^ observed larger choroidal thickness from *post mortem* human tissue in eight AD patients relative to eleven controls using histopathology, while Robbins et al.^19^ found larger total choroidal area and total vessel area in AD compared to controls in enhanced‐depth OCT. However, these measurements were reported in pixel space. Moreover, the authors observed significantly lower choroidal thickness in their sample, but these distances were measured without accounting for potential choroidal curvature. Thus, where our results fit into the disease trajectory of AD is yet to be determined.

In this exploratory study of asymptomatic people at increased risk of AD, we observed trends which, independent of covariates, indicate a larger retinal choroid vasculature in participants who carry the *APOE* ε4 genotype or have a FH, relative to those who do not. This reached statistical significance in total vessel area and total choroid area. However, subfoveal thickness is less robust in characterizing the choroid than area, and the relationship between CVI and vessel area/choroid area likely played a role in any significant, distributional differences observed between groups (see “Interplay of choroidal measurements” in supporting information).

Furthermore, measurement error and diurnal variation are unlikely to have had a major effect on our results. The differences in mean choroid measurements between *APOE* ε4 carriers and non‐carriers (choroidal thickness, 53 microns; total choroidal area, 0.19 mm^2^; CVI, 0.05; and total vessel area, 0.15 mm^2^) were greater than expected from approximate choroidal fluctuation due to diurnal variation (choroidal thickness, 30 microns; total choroidal area, 0.035 mm^2^; CVI, 0.015; total vessel area, 0.02 mm^2^),^45^, ^46^, ^47^, ^48^, ^49^ and lower than the threshold reported on Choroidalyzer's reproducibility for choroidal analysis on single‐line OCT B‐scans (choroidal thickness, 11.5 microns; total choroidal area, 0.05 mm^2^; CVI, 0.013).^50^ This was also the case for individuals with and without a FH (Table 3).

The ordinal models give us an indication of the size of the observed associations found in our sample and can be easily interpreted. For example, consider two males of equal age, with the same blood pressure. If the first male's total vessel area was greater by at least 0.16 mm^2^, they are on average more than two times likely to have at least one (or both) of the risk markers, assuming the comparative individual had no genetic markers, thus placing them in a higher risk group for developing later life dementia. Whether this effect exists within the general population should be investigated in cohorts designed to test this hypothesis.

Strengths of the current study include the use of reproducible and fully automatic software, Choroidalyzer, to extract measurements from the choroidal vasculature using a standardized ROI. Further, the software reports values in physical units rather than pixel units, aiding interpretability and helping mitigate spurious associations.^24^ Another strength is the uniqueness of the PREVENT Dementia cohort, which permits characterization of the ocular microvasculature in asymptomatic individuals, at increased genetic risk, and ≈ 20 years younger than most individuals in similar studies.

There were some limitations to this study. Statistical analysis was limited by low power due to the small sample size and we cannot be certain that selection bias did not play a role in our results. A lower sample size also prevented the use of mixed models, which can account for the nested structure of eyes within participants. The low sample size also limited the number of covariates. Additionally, participants were primarily White in ethnicity; therefore, the generalizability of our findings to other ethnic groups or the wider population may be limited.

Finally, our data collection protocol did not collect measurements of intra‐ocular pressure (IOP). As IOP directly affects ocular perfusion pressure (OPP), a key regulator of choroidal blood flow, it could be that the observed changes in choroidal vasculature may be attributed to variations in OPP, acting as a confounding factor independent to AD risk. Thus, while our associations could relate to mechanistic links between the choroid microcirculation and the pathogenesis of AD, more research is needed with a larger sample size and the collection of additional clinically informative variables such as IOP to better understand and account for potential confounding factors. However, the PREVENT Dementia study is ongoing, collecting longitudinal data at roughly 2‐year intervals, allowing future temporal modeling that may further validate our findings.

## 5 CONCLUSION

In this pilot study, we found that the retinal choroidal vasculature was significantly larger in individuals who were both *APOE* ε4 carriers and had an FH, compared to those with one risk factor or none. These exploratory results suggest a potential association between the retinal choroidal vasculature and AD risk, and longitudinal studies are needed to see if the retinal choroidal microvasculature may be predictive of developing future cognitive decline and later life AD. Although our findings are exploratory, they provide a foundation for future research that could lead to the development of non‐invasive biomarkers for the early detection of AD, potentially acting as a primary or secondary endpoint of future clinical trials.

## CONFLICT OF INTEREST STATEMENT

J.B.: none. S.G.: none. A.L.: awarded “Race Against Dementia” Fellowship in past 36 months. C.H.: none. M.RS.: none. G.MT.: none. C.W.R.: founder, CEO, and majority shareholder for Scottish Brain Sciences; Received consulting fees from Biogen, Eisai, mSD, Actinogen, Roche, and Eli Lilly in last 36 months; received payment for presentation‐based work by Roche and Eisai in last 36 months. B.D.: none. J.T.O.: received grant from Avid/Lilly, Merck, and Alliance Medical in last 36 months; received consulting fees from TauRx, Novo Nordisk, Biogen, Roche, Lilly, and GE Healthcare in last 36 months. S.K.: none. I.J.C.M.: none. T.J.M.: none. Author disclosures are available in the supporting information.

## CONSENT STATEMENT

All participants provided written informed consent, and the study was carried out in compliance with the Declaration of Helsinki.

## DECLARATION

The PREVENT Dementia program received multi‐site ethical approval from the UK London‐Camberwell St Giles National Health Service (NHS) Research Ethics Committee (REC reference: 12/LO/1023, IRAS project ID: 88938), which operates according to the Helsinki Declaration of 1975 (and as revised in 1983). All substantial protocol amendments have been reviewed by the same ethics committees and a favorable opinion was granted before implementation at sites. The retinal sub‐study in Edinburgh was approved by the South East Scotland Research Ethics Committee (15/SS/0146)

## Supporting information



Supporting information

Supporting information

## Data Availability

The PREVENT Dementia dataset is available to access through a data request on the study website (www.preventdementia.co.uk); on the Alzheimer's Disease Data Initiative (ADDI) platform baseline dataset DOI: https://doi.org/10.34688/PREVENTMAIN_BASELINE_700V1; Dementia Platforms UK (DPUK); and the Global Alzheimer's Association Network (GAAIN).
